# Supplementary material for: Comprehensive Annotation and Functional Exploration of MicroRNAs in Lettuce
Source: Front Plant Sci. 2021 Dec 24;12:781836. doi: 10.3389/fpls.2021.781836 (PMC8739914; doi:10.3389/fpls.2021.781836)
Supplement: Supplementary Data 1 — Results of categories 0–2 from PARE-Seq analysis (including three subfiles:1_1, 1_2, 1_3). [file Data_Sheet_10.ZIP › GSM2230754.plot/Lsa-miRN1690_Lsat_1_v5_gn_2_5001.1_59_TPlot.pdf]

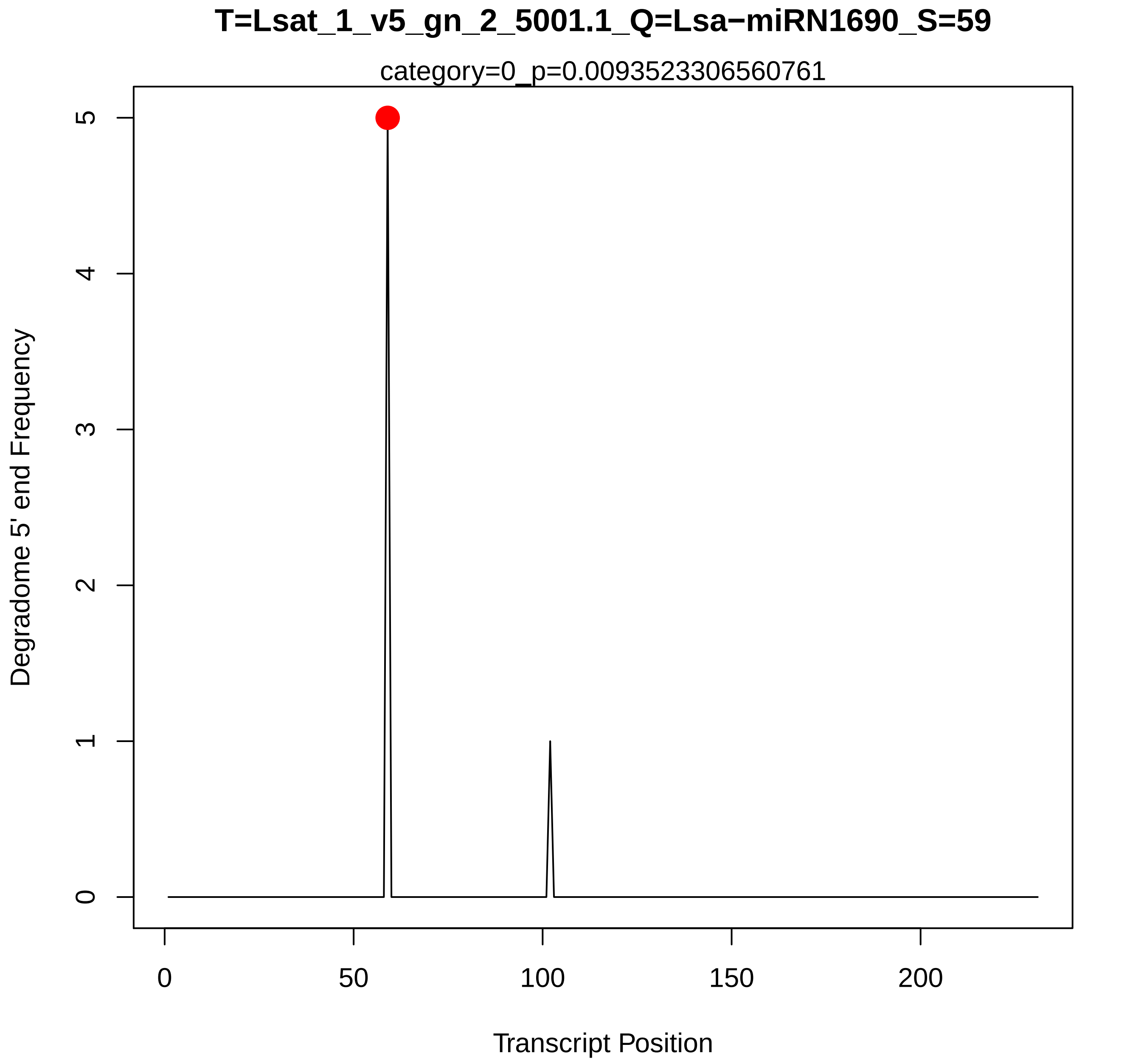

T=Lsat_1_v5_gn_2_5001.1_Q=Lsa−miRN1690_S=59
category=0_p=0.0093523306560761
Degradome 5' end Frequency
0
1
2
3
4
5
0
50
100
150
200
Transcript Position
